# Autophagy and *Mycobacterium Tuberculosis*: the role of autophagy in antimicrobial immunity and therapy

**DOI:** 10.3389/fcimb.2026.1748677

**Published:** 2026-02-16

**Authors:** Hong Lei, Junya Lan, Yanan Chen, Jie Liu, Yushan Yao, Nannan Zhou, Xiudong Ding, Ying Jiang

**Affiliations:** 1 Department of Clinical Laboratory, the 8th Medical Center of PLA General Hospital, Beijing, China; 2 Hebei North University, Zhangjiakou, Hebei, China

**Keywords:** *Mycobacterium tuberculosis*, tuberculosis, autophagy, autolysosome, autophagy genes, signaling pathways

## Abstract

Tuberculosis (TB) remains one of the most severe infectious diseases worldwide, posing a persistent and increasingly serious threat to global public health. Cellular autophagy, a highly conserved innate immune mechanism, plays a crucial role in the elimination of intracellular pathogens, regulation of immune responses, and maintenance of cellular homeostasis, making it a key focus in TB research. This review systematically summarizes the types and regulatory mechanisms of autophagy, as well as its interactions with *Mycobacterium tuberculosis* (*M. tb*), and explores the potential applications of autophagy-based host-directed therapeutic strategies. It also addresses the major challenges in current research, including the complex mechanisms by which *M. tb* evades autophagy, the selectivity and safety concerns of autophagy modulators, and the technical barriers to clinical translation. Growing evidence suggests that autophagy has emerged as a promising therapeutic target for TB, and autophagy modulators may serve as effective adjunctive therapies. Future research should further elucidate the interactions between autophagy and immunometabolic pathways, optimize the targeted delivery of autophagy activators, and verify their efficacy and safety through systematic clinical studies, thereby providing new theoretical foundations and therapeutic strategies for TB prevention and treatment.

## Introduction

*Mycobacterium tuberculosis* (*M. tb*) is one of the most lethal human pathogens known to date, capable of activating multiple cellular signaling pathways within macrophages to induce immune defense and phagocytic responses (Luo et al., 2020; Song et al., 2022). However, *M. tb* possesses sophisticated immune evasion mechanisms that effectively circumvent immune recognition and suppress anti-tuberculosis immune responses (Zeng et al., 2024). The core of its pathogenicity lies in its ability to survive and replicate within host macrophages (Morita et al., 2021). Tuberculosis, caused by *M. tb*, is an ancient and persistently prevalent infectious disease (Liu H. et al., 2023), often associated with developing countries, yet it continues to pose a severe threat to global public health. Each year, approximately 10 million new active TB cases are reported, resulting in around 1.5 million deaths (Sey and Warris, 2024). Furthermore, about one-quarter of the global population carries latent infection (Bhengu et al., 2023), while drug-resistant TB remains a critical and unresolved public health challenge.

Autophagy is present in most eukaryotic cells and is crucial for the autophagic flux, which is a dynamic process (Chen et al., 2022a). By degrading damaged cellular structures, aged organelles, misfolded proteins and other biomolecules (Bahar et al., 2022), autophagy maintains normal cellular activities. Animal cells consist of a nucleus, a cytoplasm and a cell membrane. Most biomolecules and functional organelles are located in the cytoplasm, where most cellular activities occur. This process generates a substantial amount of metabolic debris, which affects normal cellular functions. In canonical autophagy, lysosomes are the main degradative organelles, responsible for breaking down and recycling cellular components to maintain cellular homeostasis (Khan et al., 2021). Moderate autophagy can provide energy during periods of nutrient deficiency, thereby preventing oxidative damage and metabolic stress, both of which are essential for maintaining intracellular homeostasis. However, excessive autophagy can lead to metabolic stress, the over-degradation of cellular components and even cell death. Autophagy also regulates inflammatory responses, participates in antigen presentation and combats immune evasion by pathogens (Huang et al., 2023).

Further research indicates that autophagy plays a role in immune responses to TB. By promoting phagosome maturation, autophagy restricts the growth of intracellular *M. tb*, playing a significant role in defence against infection and treatment. Autophagy can directly control and eliminate *M. tb* in infected macrophages (Wu et al., 2022). However, *M. tb* can evade immune clearance in various ways (Habtamu et al., 2022). For example, it can kill immunocompromised macrophages, hinder the fusion of lysosomes with autophagosomes, prevent enzymatic degradation (Momeni et al., 2024), and cause cell necrosis. Simultaneously, it may also inhibit phagosome maturation and autophagy, reduce apoptosis, and weaken adaptive immunity. Thus, a complex ‘offensive–defensive’ relationship is formed between autophagy and *M. tb*.

This review summarises the mechanisms of action, research progress and future directions of autophagy in TB. The review focuses on the immune defence mechanisms of autophagy during *M. tb* infection, the strategies employed by the pathogen to escape the immune response, and the crucial role of autophagy in regulating the host immune response. It also explores the regulatory functions of autophagy pathways in anti-TB therapy. Additionally, it analyses recent advancements in host-directed therapies based on autophagy activation and assesses their potential to enhance anti-TB efficacy, alleviate tissue damage and address drug resistance. Finally, it points out current research challenges and deficiencies and proposes future research directions, aiming to provide theoretical foundations and new approaches for TB basic research and clinical intervention.

## Theoretical background

### Types and basic processes of autophagy

**Figure 1:**
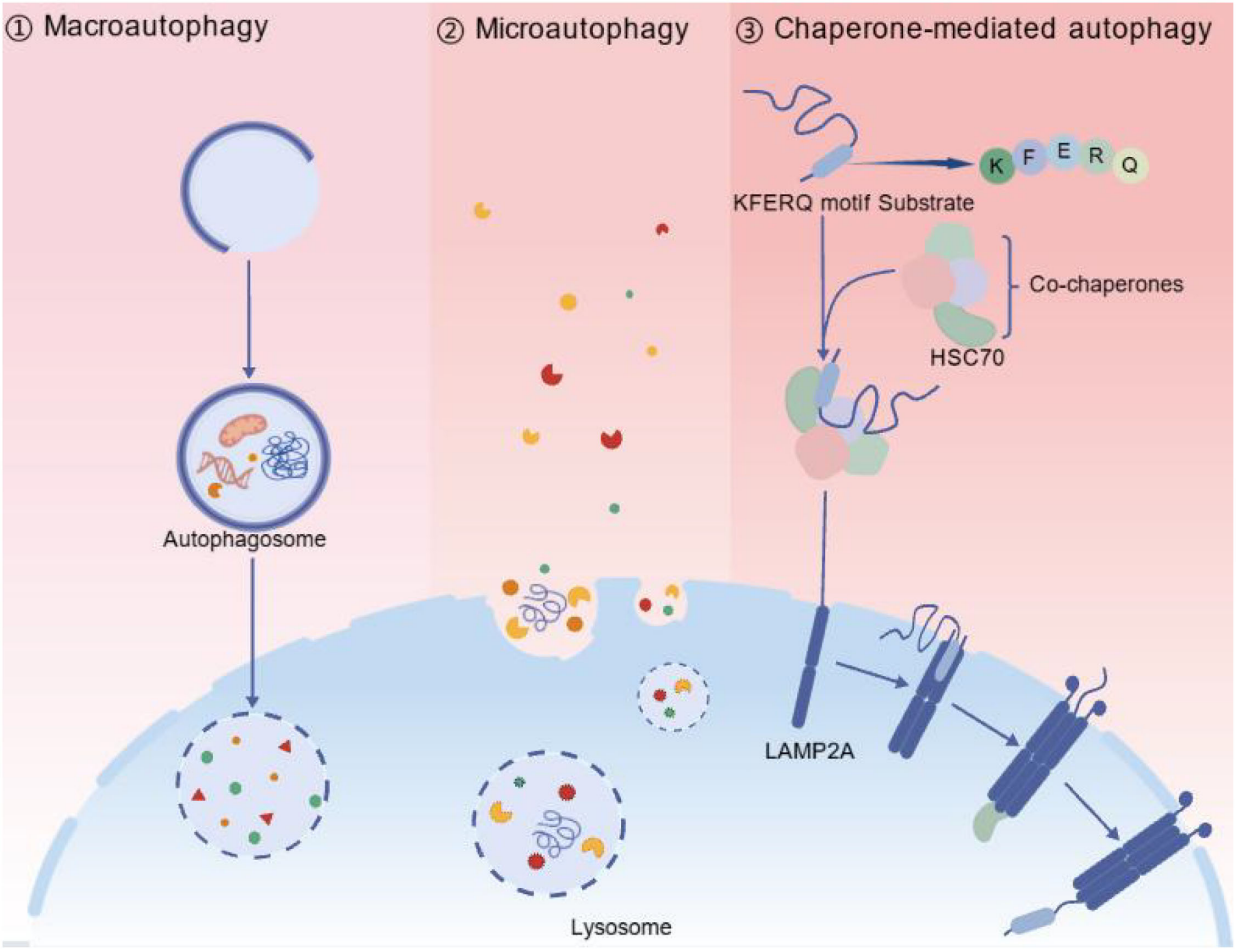
Three major types of autophagy. Autophagy can be divided into three main forms: macroautophagy, microautophagy, and chaperone-mediated autophagy (CMA). 1. Macroautophagy involves the formation of a double-membrane autophagosome that engulfs cellular materials. These subsequently fuse with lysosomes for degradation. 2. Microautophagy directly engulfs target materials through invagination or protrusion of the lysosomal membrane, followed by degradation within the lysosomal lumen. 3. Chaperone-mediated autophagy (CMA) selectively recognises substrate proteins containing a specific KFERQ-like motif. These proteins are identified and bound by the molecular chaperone HSC70 and then translocated into the lysosome via the lysosome-associated membrane protein LAMP2A, where they are degraded. These three autophagic pathways maintain cellular homeostasis by eliminating damaged or unnecessary cellular components, thereby preserving normal cellular function.

Autophagy is categorised into three types based on the pathways through which substrates enter lysosomes: macroautophagy, microautophagy and chaperone-mediated autophagy (**Figure 1**) (Mahapatra et al., 2021). Among these, macroautophagy is the most extensively studied and crucial for anti-TB.

### Macroautophagy

During nutrient deficiency, macroautophagy degrades proteins, fats, and glycogen to provide energy and metabolic substrates for cells (Lim and Murthy, 2020), preventing cell necrosis due to nutrient insufficiency. In nutrient abundance, macroautophagy clears misfolded proteins and damaged organelles, preventing their accumulation and subsequent damage, thus maintaining intracellular environmental stability. Therefore, macroautophagy balances energy sources under energy stress. The formation of autophagosomes is a dynamic process, initially forming a double-membrane structure known as a phagophore or separation membrane (Lu et al., 2022). The phagophore expands to enclose materials for degradation and eventually closes to form a complete double-membrane vesicle, the autophagosome (Zhao et al., 2022). The autophagosomal membrane may originate from intracellular membrane structures such as the endoplasmic reticulum, plasma membrane, Golgi apparatus, and mitochondria (He and Tian, 2020). Subsequently, the autophagosome traverses the cytoplasm to the lysosome and fuses with it to form an autolysosome (Sobolewski and Legrand, 2021). The lysosome contains various hydrolytic enzymes, such as proteases and lipases, which break down vesicle contents into basic metabolic products. These basic metabolic products are then released into the cytoplasm for reuse. The process of macroautophagy involves the regulation of various autophagy-related genes and signaling molecules, with its precise control being closely related to cell survival and metabolic balance and also playing a significant role in cancer, infection defense, and neurodegenerative diseases (Zhang C. et al., 2020; Zhu et al., 2022).

### Microautophagy

Compared to macroautophagy, which forms autophagosomes to enclose materials, microautophagy operates more directly. It involves morphological changes such as invagination or protrusion of the lysosomal membrane, encapsulating target materials, forming vesicles within the lysosome, and incorporating them into the lysosomal lumen for degradation, ultimately producing small molecules that are released back into the cytoplasm for cellular utilization (Yao and Shen, 2023; Li et al., 2024). This process does not require the formation of independent autophagosomes but relies on the dynamic reshaping of the lysosomal membrane to directly complete material uptake (Liu P. et al., 2023). Although less researched, microautophagy remains significant in maintaining intracellular environmental homeostasis, regulating energy metabolism, and responding to stress reactions.

### Chaperone-mediated autophagy

Chaperone-mediated autophagy is a highly selective form of autophagy that utilizes molecular chaperones to recognize specific target proteins through distinct domains and transport them to lysosomes for degradation (Dubois et al., 2020; Liu et al., 2022). Its characteristics include strong substrate specificity, precise regulatory mechanisms, structural simplicity, and independence from membrane structure formation.

In this process, heat shock homolog 70kDa protein (HSC70) recognizes proteins containing the pentapeptide sequence Lys-Phe-Glu-Arg-Gln (KFERQ) and delivers them to lysosomes for degradation (Xu and Yang, 2022). Chaperone-mediated autophagy specifically identifies cytosolic proteins containing the KFERQ-like sequence, composed of specific amino acid residues: a glutamine (Q), an amino acid lysine (K) or arginine (R), a hydrophobic amino acid phenylalanine (F), valine (V), leucine (L) or isoleucine (I), an acidic amino acid glutamate (E) or aspartate (D), and ending with a hydrophobic or basic amino acid residue. This ensures strict substrate selectivity. The exposed KFERQ sequence is precisely recognised by cytosolic HSC70, forming a stable complex between the substrate and HSC70 and marking the initiation of chaperone-mediated autophagy. This complex is then transported to the lysosomal membrane where it binds to lysosome-associated membrane protein 2A (LAMP2A) (Bai et al., 2022). LAMP2A recognises and transports the substrate-chaperone complex into the lysosome (Zarrabi et al., 2023). Under the aggregation interaction of multiple LAMP-2As, unfolded substrate proteins pass through the lysosomal membrane into the lysosomal cavity (Jia et al., 2024), where they are degraded into small molecules by proteases. After degradation, the LAMP-2A multimer disassembles into monomers for recycling (Ho et al., 2020).

### Autophagy-related genes (ATGs) and proteins and their functional significance in disease

Autophagy depends on a set of proteins encoded by autophagy-related genes, which were initially discovered in yeast. To date, over 40 ATG proteins have been identified (Abd El-Aziz et al., 2021; Gao et al., 2024). ATG proteins play crucial roles at various stages of autophagy, including induction, formation and extension of isolation membranes, maturation of autophagosomes, and fusion of autophagosomes with lysosomes. During the initiation stage, the ULK1–ATG13–FIP200–ATG101 complex acts as the primary ATG activation module, which marks the beginning of autophagosome formation (Li et al., 2020). Subsequently, a Beclin1 (ATG6)-centred ATG protein complex mediates phagophore nucleation (Wang et al., 2023), with ATG14 conferring specificity to the Beclin1 complex for canonical autophagy. The expansion of the autophagosome membrane relies on two ATG-dependent ubiquitin-like conjugation systems: the ATG12–ATG5–ATG16 complex and LC3/ATG8 lipidation (He, 2022). Within these systems, ATG7 acts as an E1-like activating enzyme, which is essential for both conjugation pathways (Li H. et al., 2022). ATG5, meanwhile, serves as a key scaffold protein that is required for the elongation and stabilisation of the autophagosomal membrane (Cui et al., 2025). Dysfunction of the aforementioned ATG proteins will directly result in impaired autophagy flux.

**Figure 2:**
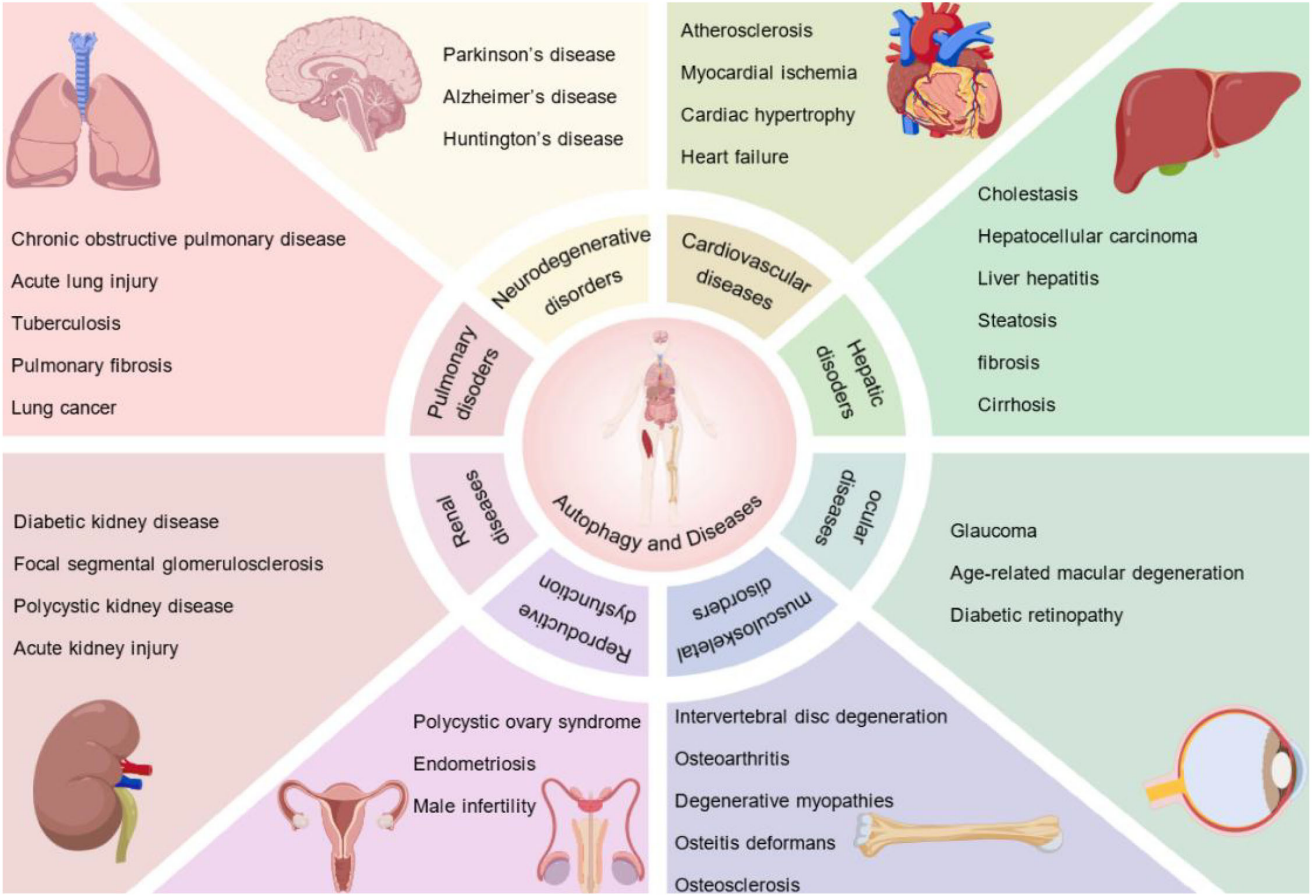
Autophagy-related diseases affecting major organ systems. Autophagy plays a role in the development and progression of various human diseases that affect multiple organ systems, including neurodegenerative, cardiovascular, pulmonary and renal disorders. Furthermore, autophagy plays a vital role in reproductive dysfunction, musculoskeletal diseases and ocular disorders. Dysregulation of autophagy contributes to the development and progression of a wide range of human pathologies.

Beyond their fundamental roles in autophagy regulation, functional abnormalities of autophagy-related genes and key proteins are closely associated with a wide range of diseases (**Figure 2**) (Zhang et al., 2023). In neurodegenerative disorders, impaired autophagy leads to defective clearance of misfolded proteins, such as tau and α-synuclein (Tian et al., 2020). In addition, heterozygous loss of Beclin1 results in autophagy deficiency and promotes tumorigenesis (Bradley et al., 2022). Autophagic homeostasis is essential for maintaining cardiovascular function and multi-organ integrity, and its disruption contributes to the development and progression of diseases affecting the liver, kidney, and respiratory system. Beyond these systems, autophagy also plays important physiological roles in regulating reproductive function, skeletal muscle metabolism, and ocular health (Chen and Lin, 2022). Collectively, autophagy-related genes and their key proteins form a multilayered regulatory network that precisely governs the dynamic process of autophagy.

### Regulation of autophagy

#### Autophagy signaling pathways

mTOR (mammalian target of rapamycin) mainly exists in two functional complexes: mTOR complex 1 (mTORC1) and mTOR complex 2 (mTORC2). As a key upstream negative regulator that initiates autophagy, mTORC1 functions by integrating signals from growth factors, amino acids, glucose, and cellular energy status (Kloska et al., 2020). When nutrients are abundant, mTORC1 is activated, thereby inhibiting autophagy. In the event of a nutrient deficiency, AMP-activated protein kinase (AMPK) becomes activated and can inhibit mTOR, thereby activating the ULK1 complex (ULK1/2, ATG13, ATG101 and FIP200) (Kong et al., 2024). The activated ULK1 complex initiates autophagy by phosphorylating downstream substrates (Chong et al., 2021). The PI3KC3 (VPS34) complex, which is composed of VPS34 (phosphatidylinositol 3-kinase), VPS15, Beclin1 (homologue of yeast Atg6) and ATG14L, catalyses the generation of phosphatidylinositol-3-phosphate (PI3P) from phosphatidylinositol (PI). This provides membrane anchoring sites for autophagosome nucleation and recruits downstream effectors such as WIPI2 and DFCP1, etc (Ye et al., 2020; Zhang et al., 2022). ATG12 is covalently bound to ATG5 via the E1-like enzyme ATG7 and the E2-like enzyme ATG10 to form the ATG12-ATG5 complex. This then binds to ATG16 to form a multimer that is localised on the autophagosomal membrane. This promotes membrane extension and closure to ultimately form the double-membrane structure of the autophagosome (García-Niño et al., 2021; Su et al., 2022). LC3, as the mammalian homolog of yeast Atg8 (Ding et al., 2022), undergoes lipidation at this stage, which serves as an important hallmark and is widely used to assess autophagic activity. The precursor LC3 (LC3-I) is exposed to its C-terminal glycine by ATG4 protease cleavage, then modified by ATG7 (E1-like enzyme) and ATG3 (E2-like enzyme), and LC3-I binds to phosphatidylethanolamine (PE) to form LC3-II (Zheng et al., 2022). LC3-II anchors to the autophagosomal membrane and cooperates with receptor proteins such as p62 (SQSTM1) and NDP52 to achieve selective recognition of cargo, ultimately leading to membrane fusion. The mature autophagosome fuses with the lysosome under the mediation of Rab7 and the SNARE complex formed by Syntaxin17, SNAP29, VAMP8, forming an autolysosome (Yang et al., 2020). The core regulatory pathway of autophagy dynamically regulates the formation of autophagosomes, substrate selection, and degradation efficiency through a multi-level signaling network. For instance, mTOR inhibitors used for cancer therapy have become a potential treatment strategy for targeted autophagy regulation. However, the effects of autophagy induced by mTOR inhibition may differ depending on tumour stage and cellular context (Zhu et al., 2023). This reflects the dual and context-dependent roles of autophagy in cancer. Future research should further analyze tissue-specific regulatory mechanisms and the crosstalk between pathways to achieve precise intervention.

In addition to the aforementioned mTOR signaling pathway, AMPK is a core energy sensor within cells and is closely related to autophagy at different stages (Gong et al., 2020). AMPK is activated when AMP levels rise and ATP levels drop, or under conditions such as oxidative stress or hypoxia, by recognising the ratio of ATP to AMP/ADP. This activates anabolic synthesis and catabolism.

**Figure 3:**
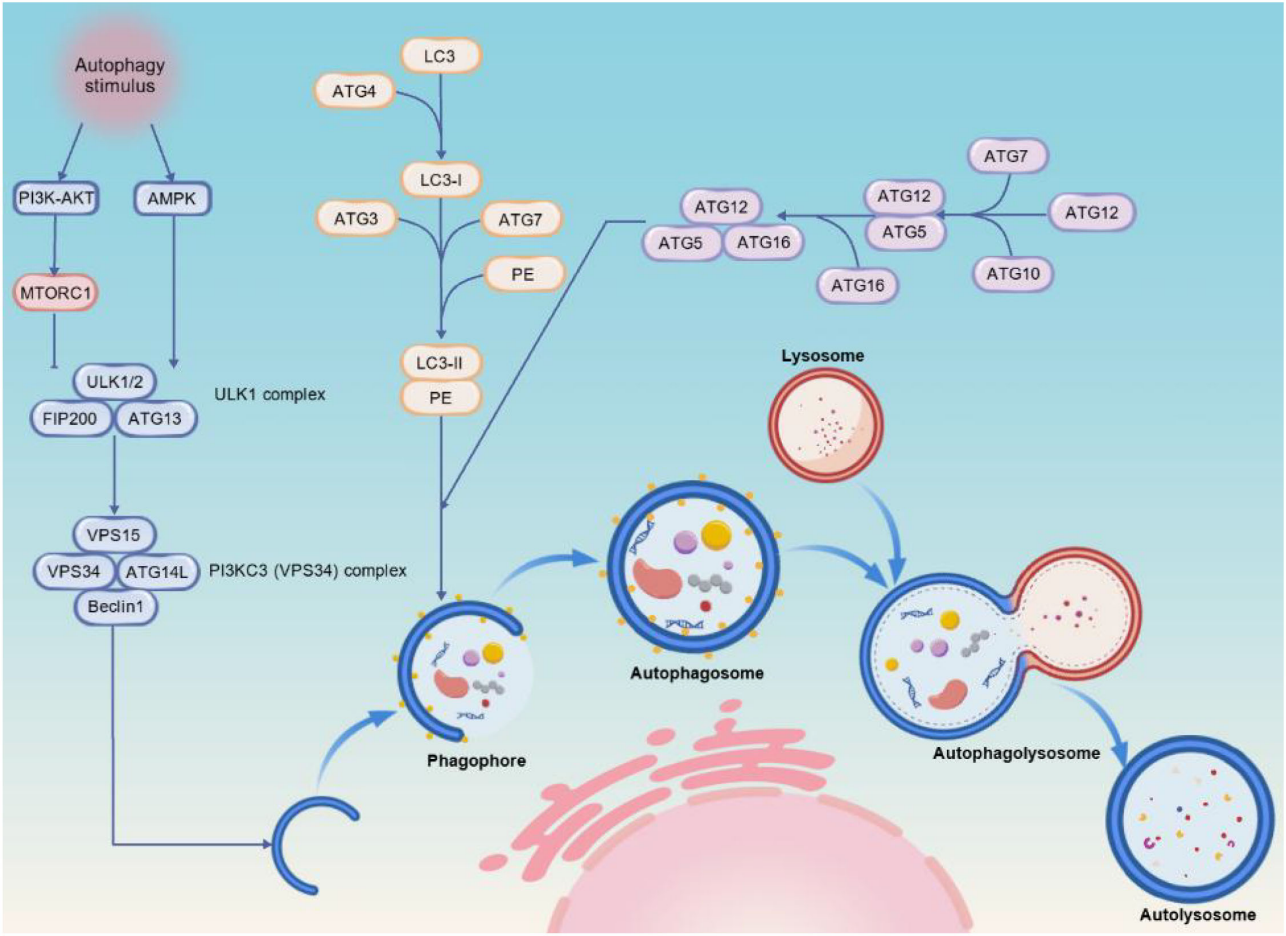
Molecular mechanisms and processes of autophagosome formation. Autophagy initiation is regulated by multiple signalling pathways, including the PI3K–AKT, AMPK and mTORC1 pathways. Inhibiting mTORC1 activates the ULK1 complex (comprising ULK1/2, ATG13 and FIP200), which then promotes the recruitment and activation of the PI3KC3 (VPS34) complex (consisting of VPS15, VPS34, ATG14L and Beclin1). This initiates phagophore formation. The ATG12–ATG5–ATG16 complex then facilitates membrane elongation and closure. LC3 undergoes lipidation (LC3-I → LC3-II) and becomes anchored to the autophagosomal membrane. The mature autophagosome then fuses with the lysosome to form an autolysosome, in which cytoplasmic components are degraded and recycled to maintain cellular homeostasis.

During the regulation of autophagy, AMPK can directly phosphorylate and activate the ULK1 complex to initiate autophagy. It can also phosphorylate the Raptor subunit of the mTORC1 complex to inhibit the mTORC1 pathway and lift its inhibition of ULK1 (Bi et al., 2025). Furthermore, AMPK can indirectly promote autophagy by regulating transcription factors such as TFEB and FOXO3, which control the expression of multiple autophagy-related genes (Jang et al., 2023). In TB, AMPK-mediated autophagy helps to clear *M. tb*, thereby enhancing the host’s cellular autonomous immune defence. Through multi-target regulation and energy sensing, AMPK plays a significant role in regulating autophagy, maintaining cellular homeostasis and resisting pathological damage. AMPK also participates in the regulation of various energy metabolic pathways. It promotes fatty acid oxidation and glycolysis and inhibits the synthesis of liver fat, proteins and muscle glycogen, thereby helping cells to maintain their energy supply under metabolic stress (Lodge et al., 2023). Other pathways, such as the PI3K-AKT pathway and the p53 pathway, also participate in the regulation of autophagy. In-depth analysis of the mechanisms of the autophagy signalling pathway can help to provide new targets for various diseases (**Figure 3**), especially for host-directed autophagy regulation therapy, which has application prospects.

#### Induction of autophagy by cellular stress

Various stresses, such as nutrient deficiency, hypoxia, inflammation, reactive oxygen species (ROS) and bacterial infection, can induce autophagy pathways (Montazersaheb et al., 2022). Under conditions of nutrient abundance, the mTOR kinase is activated, which inhibits autophagy by phosphorylating the ULK1/2 initiation factors. Under conditions of nutrient deficiency, however, energy insufficiency leads to an increased AMP/ATP ratio, activating AMPK, which inhibits mTORC1 and simultaneously activates the ULK1 complex through parallel mechanisms, thereby initiating autophagy (Ning et al., 2024). ROS, a marker of cellular oxidative stress, can activate autophagy directly to eliminate the source of oxidative stress and prevent cellular damage (Echavarria-Consuegra et al., 2019). ROS can regulate autophagy at multiple mechanistic levels. Under conditions of oxidative stress, ROS activate AMPK, which promotes the ULK1-mediated initiation of autophagy (Zheng et al., 2025). ROS can also modulate the formation of autophagosomes through redox-dependent modifications of key autophagy-related proteins, such as ATG4, thereby facilitating LC3 lipidation (Deng et al., 2017). Meanwhile, the activation of stress-responsive kinases induced by ROS, such as c-Jun N-terminal kinase(JNK) and p38 (Akhigbe and Ajayi, 2021), may further regulate the autophagic process in a cell context–dependent manner.

### Interactions between *Mycobacterium Tuberculosis* and host cells

*M. tb* employs multiple strategies to infect host cells and disrupt their normal activities, thereby evading immune clearance and achieving prolonged survival within them (Chen et al., 2021). In-depth research into the dynamic interaction between *M. tb* and host cells is crucial for developing new anti-TB treatments and inhibiting *M. tb* survival within host cells. This makes the study of the interaction between *M. tb* and host cells highly significant.

### Macrophages defending *Mycobacterium Tuberculosis*

The pathogenicity of *M. tb* largely depends on its ability to infect the host and evade the immune response of host macrophages, including inducing macrophages towards an immunosuppressive phenotype, promoting macrophage foam cell formation, and manipulating cell apoptosis and autophagy. Macrophages, as the first line of defense of the host immune system against *M. tb* invasion, possess multiple functions such as bactericidal activity, phagocytosis, antigen presentation, and immune regulation (Wang et al., 2020). (1) Pathogen recognition and phagocytosis: *M. tb*, based on cell wall components such as lipoarabinomannan (LAM), phosphatidylinositol mannoside (PIM), and other outer membrane molecules, binds to macrophage membrane surface receptors such as mannose receptor (MR), complement receptor CR3, etc., thereby mediating phagocytosis (Wang et al., 2022). Subsequently, the phagosome fuses with the lysosome to form a phagolysosome, killing the pathogen through an acidic environment and hydrolytic enzymes. (2) Intracellular bactericidal and clearance: Macrophages can produce ROS, reactive nitrogen species (RNS), and various lysosomal enzymes to collectively clear *M. tb* (Shi et al., 2023). (3) Pro-inflammatory response and cytokine release: Upon recognizing *M. tb*, macrophages release inflammatory factors such as IL-6, TNF-α, etc., to regulate the cell’s nonspecific immunity against *M. tb*. (4) Antigen processing and presentation: Macrophages present processed antigen fragments to CD4^+^ T cells via MHC-II molecules, thereby inducing an immune response (Xu et al., 2022). (5) Granuloma formation and maintenance: At the site of *M. tb* infection, differentiated epithelioid cells, multinucleate macrophages, dendritic cells, B cells, and T cells collectively form granulomas to limit pathogen dissemination (HaileMariam et al., 2021). (6) Inducing programmed cell death and affecting the disease course: *M. tb* infection of macrophages can induce multiple forms of cell death, including apoptosis, necroptosis, unregulated necrosis, and pyroptosis. *M. tb* modulates these pathways by inhibiting host-protective apoptosis while promoting necrotic and pro-inflammatory forms of cell death, thereby influencing the host disease course and facilitating bacterial infection and transmission.

### Mechanisms of immune evasion by *Mycobacterium Tuberculosis*

*M. tb* possesses various immune evasion mechanisms that enable it to survive within the host long-term and cause chronic infections. (1) Preventing fusion of phagosomes with lysosomes: After *M. tb* enters macrophages, it can secrete effector proteins that prevent the fusion of phagosomes with lysosomes, thereby avoiding subsequent elimination or degradation (Quan et al., 2022). (2) Antioxidant and anti-nitrogen intermediates: *M. tb* reduces the production of RNS and ROS intermediates to resist oxidative damage. (3) Inhibiting macrophage apoptosis and autophagy: *M. tb* keeps infected macrophages from undergoing apoptosis, prolonging the survival time of the pathogen within the cells (Mo et al., 2024). At the same time, it can regulate the fusion of autophagosomes with phagosomes, weaken lysosomal function, and inhibit cellular autophagy. (4) Suppressing antigen presentation: *M. tb* inhibits the expression of MHC-II molecule-related transcription factors and their molecules, weakening antigen presentation functions (Korbonits et al., 2022). (5) Establishing latent infection within granulomas: The host forms granulomas to suppress bacteria, while *M. tb* can enter a dormant state within the oxygen-deprived and nutrient-deficient granulomas, enhancing resistance to the environment and the host. Additionally, *M. tb* can employ other immune evasion mechanisms to maintain long-term survival under the pressure of the host immune system and drug treatment.

### PE/PPE and PE_PGRS protein families involved in hijacking host autophagy

*M. tb* employs multi-level strategies to hijack the host autophagy pathway, thereby sustaining its intracellular survival and long-term infection. *M. tb* reduces pathogen degradation and suppresses antigen presentation and inflammatory responses by obstructing autophagosome maturation and lysosomal fusion, inhibiting autophagy initiation signals, and modulating host signaling pathways (Sit et al., 2020; Liu et al., 2020, Liu D. et al., 2023). These mechanisms facilitate bacterial evasion of immune defenses while also weakening the host’s adaptive immune response.

The PE/PPE/PGRS protein family is unique to the *M. tb* genome and accounts for around 10% of its coding capacity (Sharma et al., 2021). It is also closely associated with immunomodulation and virulence. Recent studies have demonstrated that PE_PGRS47 and PE_PGRS20 can directly interact with the host small GTPase Rab1A (Strong et al., 2021). This blocks the initiation of autophagy (Guallar-Garrido and Soldati, 2024), reduces the recruitment of autophagy-related proteins and suppresses the autophagic process, thereby enhancing bacterial survival within macrophages. These specific PE_PGRS proteins act as effectors that directly target core regulatory components of host autophagy, such as Rab1A and ULK1. These effectors impair autophagy and indirectly reduce pro-inflammatory cytokine production and antigen presentation efficiency (De Maio et al., 2020). In addition, the PPE51 protein has been shown to indirectly inhibit autophagy by suppressing the TLR2–ERK1/2 signalling pathway (Veerapandian et al., 2024). PPE51 expression attenuates host responsiveness to autophagy-inducing stimuli and interferes with TLR 2 signalling, leading to reduced activation of downstream kinases and consequently weakening autophagy-mediated antibacterial responses (Strong et al., 2022). Other PE/PGRS proteins, such as PE_PGRS41, have been reported to indirectly suppress autophagy-related defence responses by inhibiting host immune functions, including the production of cytokines and the activation of cell death pathways (Bo et al., 2023). This promotes the survival of the bacterium within host cells.

In summary, *M. tb* uses a variety of immunomodulatory proteins, including PE/PGRS and PPE, to precisely control host autophagy. This regulation involves the direct targeting of core autophagy molecules and signalling complexes, as well as the interference of pattern recognition receptor signalling and downstream inflammatory responses.

## Current research status

### Immune role of autophagy in tuberculosis infection

#### Function of autophagy in phagosome maturation and bacterial killing

Following *M. tb* infection, autophagy plays a crucial role in immune defence, particularly in phagosome maturation and bacterial killing. Upon activation by signals such as Dectin-1 and TLR, the phagosomal membrane can recruit the autophagy protein LC3-II. Atg5, Atg7 and other related proteins then promote the recruitment of maturation markers such as Rab7, thereby accelerating phagosome maturation and promoting its fusion with lysosomes to enhance the bactericidal effect (Dumas and Knaus, 2021; Pang et al., 2022). Furthermore, autophagy can enhance the effects of ROS and RNS, resulting in multi-level bactericidal actions. It can also regulate antigen processing and presentation, as well as cytokine secretion, thereby modulating innate and adaptive immunity (Chen et al., 2022a). Overall, autophagy promotes pathogen encapsulation, phagosome maturation, fusion with lysosomes and bactericidal degradation through its synergistic action with phagosomes. However, *M. tb* has developed strategies to hijack the innate immune system by blocking phagosome maturation and fusion with lysosomes, enabling cytoplasmic escape and inhibiting autophagy (Zhou et al., 2023).

#### Autophagy’s involvement in inflammatory regulation and apoptosis

Studies indicate that autophagy plays a dual role in regulating host inflammatory responses and apoptosis. Autophagy reduces the activation of inflammatory bodies and inflammatory responses by clearing damaged mitochondria and ROS, and by degrading NLRP3 inflammatory bodies. This inhibits excessive inflammation and maintains immune homeostasis (Zhu and Liu, 2022). At the same time, autophagy can regulate the production and secretion of inflammatory factors, playing an important role in anti-infective immunity. Additionally, autophagy exerts a bidirectional regulatory effect on apoptosis, the specific manifestation of which depends on cell type, stimulus type and duration (Zudeh et al., 2023). On the one hand, autophagy can inhibit apoptosis by clearing pro-apoptotic factors and regulating the Bcl-2 protein family and other mechanisms. On the other hand, under certain special circumstances, the autophagy process can transform into a pro-apoptotic signal, thereby activating apoptosis. Key regulatory proteins in the processes of autophagy and apoptosis can directly or indirectly regulate both processes, but the mechanisms of their interconversion and the impact of related regulatory proteins on cells still require further study. Therefore, an imbalance in the regulation of autophagy in inflammation and apoptosis is involved in the development of various diseases.

### Ubiquitination-mediated xenophagy mechanism

During *M. tb* infection, host cells can selectively recognize and eliminate intracellular pathogens through xenophagy. The bacteria rely on the ESX-1 secretion system to mediate phagosomal damage, cytosolic release, and ubiquitination (Kim et al., 2019). Bacterial DNA is recognized via STING, which further recruits autophagy adaptor proteins such as NDP52 and p62, thereby activating xenophagy (Ren et al., 2022). In this process, Smurf1 and Parkin play critical roles: Smurf1, an E3 ubiquitin ligase, primarily mediates k48-linked ubiquitination, whereas Parkin mainly participates in k63-linked ubiquitination (Campos et al., 2022). Together, they coordinate the tagging of intracellular pathogens and promote selective autophagy. Multiple studies have shown that the ubiquitin ligase Smurf1 is not only essential for anti-*M. tb* immunity but also contributes to the regulation of pulmonary inflammation and the activation of autophagy pathways, making it an indispensable regulator of host defense against tuberculosis.

### Non-canonical autophagy

LC3-associated phagocytosis (LAP) is a non-canonical form of autophagy that originates from invaginations in the plasma membrane, forming single-membrane phagosomes that are modified by the lipid LC3 (Judith et al., 2023). *M. tb* can activate TLRs, which, upon recognising pathogens, activate downstream signalling pathways and recruit PI3KC3 (VPS34) complex to single-membrane phagosomes. This catalyses the generation of PI3P, which, together with Rubicon, stabilises the NOX2 complex and drives ROS production (Wong et al., 2018; Nah et al., 2021). Various ATG proteins are then recruited to the phagosomal membrane, catalysing the formation of LC3-II and its surface modification. Ultimately, the phagosome fuses with the lysosome to complete the clearance of the pathogen. The LAP pathway can effectively prevent *M. tb* from inhibiting lysosomal maturation.

**Figure 4:**
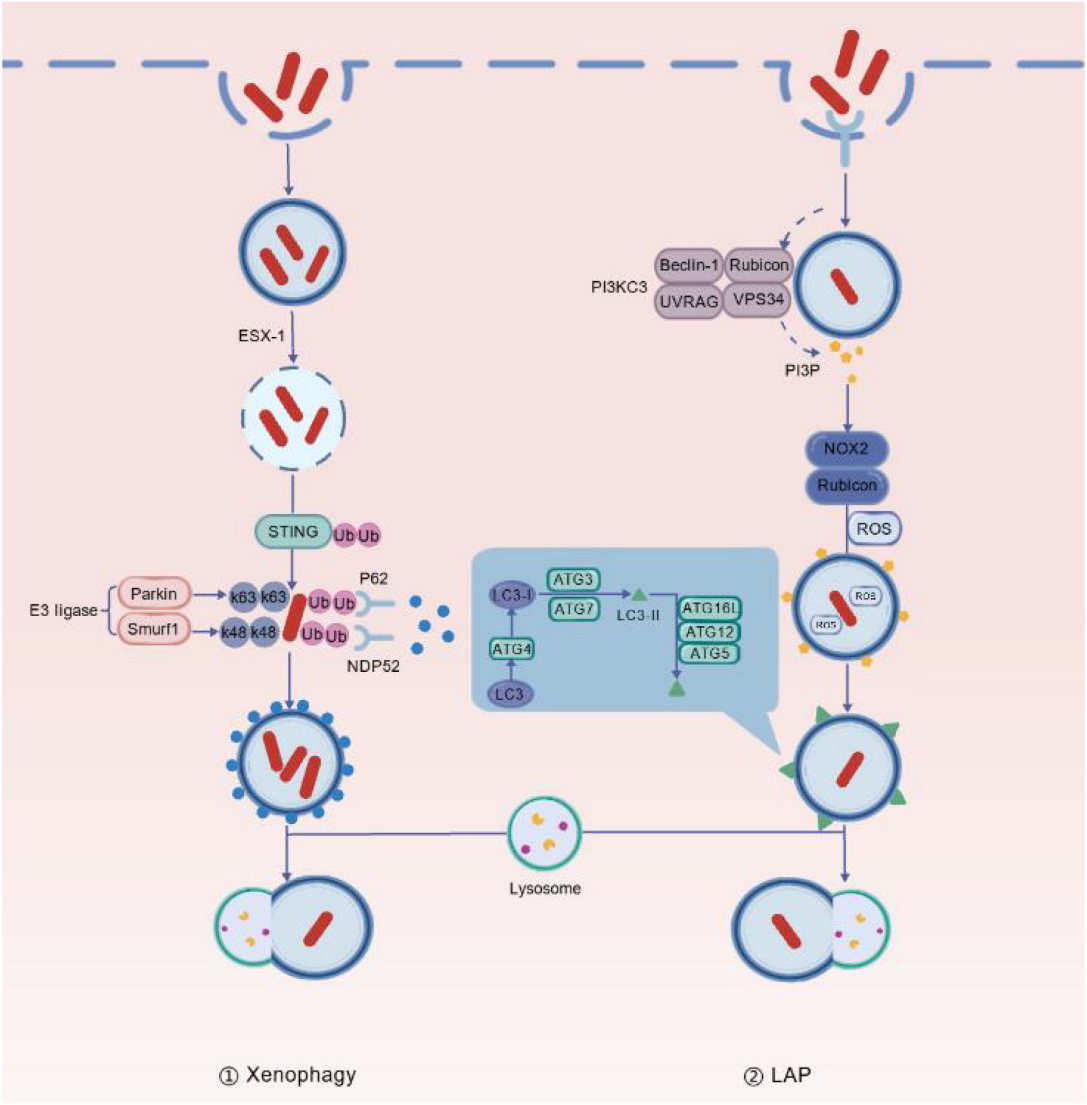
Xenophagy and LC3-associated phagocytosis (LAP) in host defense against *M. tb*. (1) During *M. tb* infection, bacteria enter the cytosol via the ESX-1 secretion system in a process known as xenophagy. Subsequently, bacterial DNA is recognised by STING, triggering ubiquitination. E3 ubiquitin ligases such as Parkin and Smurf1 then mediate distinct types of ubiquitin chains, recruiting autophagy receptors such as NDP52 and p62 and promoting the formation of autophagosomes and the degradation of bacteria. (2) LAP (LC3-associated phagocytosis) involves the direct conjugation of LC3 to single-membrane phagosomes and can be activated via multiple receptor signals, including Toll-like receptors (TLRs). During this process, the PI3KC3 (VPS34) complex (comprising VPS34, Beclin-1, UVRAG and Rubicon) is recruited to the phagosomal membrane to generate PI3P. Meanwhile, the NOX2 complex produces reactive oxygen species (ROS), coordinating the recruitment of autophagy-related proteins and promoting the attachment of lipidated LC3-II to the membrane.

In summary, xenophagy and LC3-associated phagocytosis (LAP) function as core defense mechanisms against *M. tb*, jointly maintaining host anti-infective homeostasis (**Figure 4**). Elucidating their regulatory mechanisms provides important theoretical support for developing new strategies for tuberculosis prevention and treatment.

### Autophagy and host-directed therapy

#### Autophagy inducers

Autophagy plays a significant role in the host’s immune response against *M. tb*. Autophagy inducers can regulate autophagy by modulating intracellular signaling pathways.

Rapamycin and everolimus are both mTOR inhibitors, primarily activating autophagy by inhibiting the mTORC1 signaling pathway, thereby relieving its inhibition of autophagy and activating the ULK1 complex to initiate autophagy (Shi et al., 2021). mTORC1 is sensitive to rapamycin, while mTORC2 is relatively resistant to the inhibitory induced by rapamycin, but long-term treatment with rapamycin can inhibit mTORC2 activity (Maiese, 2023). In TB treatment, it is necessary to balance its promotion of autophagy and its impact on the immune system. High doses of everolimus have immunosuppressive effects, while low doses can enhance immunity (Lin et al., 2025). Rapamycin and everolimus can promote the formation and maturation of macrophage autophagosomes, enhancing the clearance of intracellular *M. tb*, often serving as adjunctive strategies in TB treatment.

Among host-directed strategies known to modulate autophagy, vitamin D signaling has been one of the earliest and most extensively studied pathways in TB. Vitamin D is a fat-soluble steroid hormone that plays a key role in combating *M. tb*. Calcitriol, the active metabolite of vitamin D3 (1,25-dihydroxyvitamin D3), is crucial for regulating calcium and phosphorus metabolism in the body (Zhang Y. et al., 2020). Vitamin D3 can regulate the host defence system, the inflammatory response, mucosal immunity, the cell cycle and apoptosis by binding to the vitamin D receptor (VDR) within macrophages (Lopez et al., 2021), thereby triggering the expression of antimicrobial peptides and promoting the maturation and fusion of autophagosomes and lysosomes. Binding vitamin D3 to VDR can also promote autophagy activation by increasing free calcium levels in the cytoplasm, enhancing Beclin1 activity and inhibiting the mTOR signalling pathway (Li A. et al., 2022). Other autophagy inducers are also expected to bridge the gap between basic research and clinical application, providing new possibilities for TB treatment.

### Possibilities and challenges of antibiotics and autophagy combination therapy

Research indicates that antibiotics can induce autophagy and enhance the bactericidal effect. First-line anti-TB drugs such as isoniazid and pyrazinamide activate autophagy in infected macrophages, thereby improving their ability to kill *M. tb*. In mouse models, a combination of clofazimine and rapamycin has been shown to significantly reduce the load of drug-resistant TB bacteria and improve pulmonary pathology. This combination also induces the production of multifunctional central memory T cells, thereby enhancing the host’s immune response (Singh et al., 2023). Combining vitamin D3 and phenylbutyrate promotes LC3-II expression, enhances autophagosome formation and improves the bactericidal activity of macrophages (Rao Muvva et al., 2021). Furthermore, certain small molecule compounds have been shown to limit the survival of *M. tb* in macrophages by enhancing the autophagy pathway (Berton et al., 2022).

Combining antibiotics with autophagy regulation agents is still challenging. Some autophagy activators induce autophagy and enhance antibacterial activity at low concentrations, but cause cytotoxicity and cell death at high concentrations (Peláez Coyotl et al., 2020). Furthermore, some autophagy activators interact pharmacokinetically with first-line anti-TB drugs, thereby affecting therapeutic efficacy; for example, the metabolism of rapamycin is impacted by rifampicin. Individual differences may also affect the activation of autophagy and therapeutic outcomes; therefore, treatment strategies must be tailored to the patient. Combining antibiotics with autophagy modulators shows great promise for TB treatment, and future research should explore the mechanisms of autophagy in anti-TB treatment.

### Research progress in host-directed therapy strategies

Host-directed therapy (HDT) is a novel adjunctive treatment approach that modulates host immune responses, promotes the production of antimicrobial peptides, autophagy, and other macrophage effector mechanisms, alters the inhibitory mechanisms of pulmonary inflammation and matrix destruction (Wallis and Hafner, 2015), enhances the host ‘s innate and acquired immunity, assists in clearing pathogens, reduces lung tissue damage caused by inflammatory responses, shortens treatment duration, and improves treatment outcomes. HDT provides new methods for the treatment of drug-resistant TB and hosts with weakened immune function. Numerous studies have shown that various known drugs have the potential for HDT, such as vitamin D, which kills intracellular *M. tb* through the production of antimicrobial peptides; rapamycin, which inhibits the growth of *M. tb* in macrophages and reduces the replication of *M. tb* in the lungs by activating autophagy; statin drugs have immunomodulatory and anti-inflammatory properties and can inhibit the mTOR signaling pathway, promoting cellular autophagy and phagosome maturation (Hassanpour et al., 2019), among which simvastatin exerts its anti-TB effect by regulating cholesterol metabolism. Although HDT is still in the preclinical stage, it provides a new treatment approach for TB, especially drug-resistant TB and immunocompromised populations, laying a theoretical foundation for individualized treatment plans that combine antibacterial and host regulation.

## Research challenges and controversies

### Incomplete understanding of *Mycobacterium Tuberculosis*’s autophagy evasion mechanisms

Although studies have shown that *M. tb* can evade host autophagy clearance through various mechanisms, such as interfering with autophagy signalling pathways and inhibiting the fusion of autophagosomes with lysosomes, the specific mechanisms remain unclear. Furthermore, the question of whether *M. tb* employs different autophagy evasion strategies during latent and active TB infection is a topic that has not been widely studied. Therefore, developing a comprehensive understanding of the dynamic interaction between *M. tb* and autophagy could offer new perspectives for clinical treatment.

#### Selectivity and safety issues of autophagy modulators

Autophagy involves multiple signalling pathways and a large number of related genes. There are differences in the expression and activity of autophagy-related molecules across different tissues and cells. Modulators may act on multiple signalling pathways and affect metabolic processes. The effects of autophagy modulators may be altered by cellular physiological status and microenvironmental differences. Furthermore, long-term activation of autophagy can result in the excessive degradation of cellular components, leading to cellular damage, metabolic disorders or even cell death (Chen et al., 2024). Inhibiting autophagy can result in the accumulation of proteins and damaged organelles, which can impair heart function (Zhang et al., 2025).

#### Technical issues in clinical application translation

Although autophagy has shown significant therapeutic potential in various diseases, its clinical application still faces many challenges. As it is a highly dynamic, multi-stage process, accurately detecting autophagy flux and assessing the effects of drugs on its regulation remains difficult (Fan et al., 2022). Although some key regulatory factors have been identified, much remains unknown about the regulatory network, including the downstream targets of regulatory factors, the presence of complementary phosphatases, and the crosstalk between different regulatory pathways. These uncertainties make clinical translation more difficult.

## Conclusions and prospects

Autophagy is an important innate immune mechanism that enables the host to defend itself against *M. tb* infection. It has been widely confirmed to play a central role in inhibiting pathogen survival, regulating inflammatory responses and maintaining immune homeostasis. As related molecular mechanisms are gradually revealed, autophagy is also being considered as a potential target for the prevention and treatment of TB. Autophagy modulators are expected to complement traditional anti-TB treatment, enhancing host immunity, addressing drug-resistant TB and shortening the treatment course. Looking ahead, research on TB-related autophagy should focus on the intersection between autophagy and immune metabolic pathways to clarify their dynamic regulatory networks and accelerate technological breakthroughs in the structural optimisation, selectivity enhancement and lung-targeted delivery of autophagy activators. Systematic preclinical validation and long-term follow-up studies will facilitate the safe, efficient and precise clinical translation of strategies for regulating autophagy in the prevention and treatment of TB, offering new approaches to TB therapy.

## Glossary

TB: Tuberculosis
*M. tb*: *Mycobacterium tuberculosis*
HSC70: heat shock homolog 70kDa protein
Lys: Lysine
Phe: Phenylalanine
Glu: Glutamate
Arg: Arginine
Gln: Glutamine
LAMP2A: lysosome-associated membrane protein 2A
CMA: Chaperone-mediated autophagy
ATGs: Autophagy-related genes
mTOR: mammalian target of rapamycin
mTORC1: mTOR complex 1
mTORC2: mTOR complex 2
AMPK: AMP-activated protein kinase
VPS34: Vacuolar Protein Sorting 34
VPS15: Vacuolar Protein Sorting 15
PI3P: phosphatidylinositol-3-phosphate
PI: phosphatidylinositol
WIPI2: WD Repeat Domain, Phosphoinositide Interacting 2
DFCP1: Double FYVE-Containing Protein 1
LC3: Microtubule-Associated Protein 1 Light Chain 3
LC3-1: Microtubule-Associated Protein 1 Light Chain 3-I
PE: phosphatidylethanolamine
LC3-II: Microtubule-Associated Protein 1 Light Chain 3-II
Rab7: Ras-related protein Rab-7
AMP: Adenosine monophosphate
ATP: Adenosine triphosphate
ADP: Adenosine diphosphate
TFEB: Transcription factor EB
FOXO3: Forkhead box O3
ROS: reactive oxygen species
JNK: c-Jun N-terminal kinase
RNS: reactive nitrogen species
LAM: lipoarabinomannan
PIM: phosphatidylinositol mannoside
MR: mannose receptor
IL-6: Interleukin-6
TNF-α: tumor necrosis factor-α
MHC-II: major histocompatibility complex class II
TLR: Toll like receptors
NLRP3: NOD-like receptor thermal protein domain associated protein 3
LAP: LC3-associated phagocytosis
NOX2: NADPH oxidase 2
VDR: vitamin D receptor
HDT: Host-directed therapy;
